# Serum microRNA-122 and Wisteria floribunda agglutinin-positive Mac-2 binding protein are useful tools for liquid biopsy of the patients with hepatitis B virus and advanced liver fibrosis

**DOI:** 10.1371/journal.pone.0177302

**Published:** 2017-05-05

**Authors:** Masato Nakamura, Tatsuo Kanda, Xia Jiang, Yuki Haga, Koji Takahashi, Shuang Wu, Shin Yasui, Shingo Nakamoto, Osamu Yokosuka

**Affiliations:** 1 Department of Gastroenterology and Nephrology, Chiba University, Graduate School of Medicine, Chiba, Japan; 2 Department of Molecular Virology, Chiba University, Graduate School of Medicine, Chiba, Japan; 3 Department of Internal Medicine, Japan Community Health Care Organization Funabashi Chuo Hospital, Funabashi, Japan; Saint Louis University, UNITED STATES

## Abstract

**Background:**

Noninvasive methods to accurately and conveniently evaluate liver fibrosis are desirable. MicroRNA (miR) is one of the candidates. MiRs are small RNAs consisting of 19–25 nucleotides that negatively regulate many target genes at transcriptional levels. Recently, many researchers have focused on circulating miRs in the blood stream as biomarkers. Hepatic miR-122 has been reported to have an association with viral replication and hepatic fibrosis in chronic hepatitis B virus (HBV) and hepatic C virus (HCV) infection.

**Methods:**

We measured serum miR-122 levels in HBV- and HCV-infected patients confirmed with liver biopsy. We also investigated a novel liver fibrosis marker Wisteria floribunda agglutinin-positive Mac-2 binding protein [WFA(+)-M2BP]. We evaluated the diagnostic usefulness of these markers in hepatic fibrosis and inflammation of patients with chronic viral infection.

**Results:**

The serum miR-122 levels of HBV-infected patients were higher than those of the control subjects. In HBV-infected patients, the serum miR-122 levels of patients with advanced liver fibrosis were significantly lower. Serum WFA(+)-M2BP was significantly higher dependent on both the staging of fibrosis and the grading of inflammatory activity in patients with both HBV and HCV infection. We also observed that higher serum WFA(+)-M2BP levels augmented the prediction of advanced liver fibrosis among HBV-infected patients with lower serum miR-122 levels.

**Conclusions:**

A lower serum miR-122 level is a useful predictor of advanced liver fibrosis in HBV-infected patients. Serum WFA(+)-M2BP could predict liver fibrosis in both HBV and HCV infection. The combination of these markers may result in the more accurate evaluation of liver fibrosis in HBV infection.

## Introduction

The presence of liver cirrhosis is an independent predictor of hepatocellular carcinoma development in chronic hepatitis B virus (HBV) [1] and hepatitis C virus (HCV) infection [2]. It is important to evaluate the staging of liver fibrosis in clinical daily practice when seeing patients with chronic liver diseases. Liver biopsy is a cornerstone for the histological assessment of the liver in the evaluation and management of patients with liver disease [3], although there are some complications and technical issues such as the false-negative sampling error [4]. A liver biopsy usually requires patients to be hospitalized. For these reasons, non-invasive methods to evaluate liver fibrosis have been developed, and easier methods are urgently needed [1,2].

Exosomes are a type of extracellular vesicle. They are derived from the budding of the inner endosomal membrane, form multivesicular bodies and fuse with the plasma membrane. Exosomes include proteins, microRNAs (miR) and viruses [5]. MiRs are small noncoding RNA molecules of 19–25 nucleotides and regulate gene expression at post translational steps. The liver has a numerous endogenous miRs that play important roles in the regulation of biological processes such as cell proliferation and liver fibrosis [6]. MiRs circulate in a cell-free form in body fluids including serum, mostly in exosomes and protein-RNA complexes [7]. We and others have also reported an association between HBV infection and several miRs [8–10]. Attention should also be paid to serum miRs as liquid biopsy markers [11].

MiR-122 is a miR that is abundant in liver. Mir-122 stimulates translation of HCV RNA [12] and is one of the therapeutic targets [13]. An association between miR-122 and HBV infection, including HBV replication [14] and therapeutic effects [15], has also been reported.

Wisteria floribunda agglutinin-positive Mac-2 binding protein [WFA(+)-M2BP] is a newly recognized fibrosis marker that was developed as a marker for HCV infection in Japan [16,17]. M2BP is a heavily N-glycosylated glycoprotein and is secreted as a ligand of galectin-3 (Mac-2) [18]. Changes in the quality and quantity of M2BP produced are observed during the progression of fibrosis, and these are induced by changes in N-glycosylation [16,17].

In the present study, we measured serum miR-122 and WFA(+)-M2BP in liver biopsy-proven HBV and HCV patients. We report that the combination of these non-invasive markers is useful a diagnostic tool for liver fibrosis in HBV-infected patients. These results could shed new light on non-invasive diagnosis in patients with HBV infection and advanced liver fibrosis.

## Patients and methods

### Patients

Ninety-one patients with chronic HBV infection and 108 patients with chronic HCV infection, who received liver biopsy between January 1996 and November 2012 at the Department of Gastroenterology, Chiba University Medical School Hospital, Chiba, Japan were consecutively enrolled in this study. All patients were treatment-naïve. Twenty-three control subjects without liver disease were also enrolled. Sera were stored at -20°C until testing at Chiba University, Graduate School of Medicine. This study was approved by the ethics committee of Chiba University, Graduate School of Medicine (No. 1241/1841). This study protocol conformed to the ethical guidelines of the Helsinki Declaration. Participation in the study was posted at our institutions. Written informed consent for liver biopsy was obtained from all patients. Baseline characteristics are listed in Table 1.

**Table 1:** Baseline characteristics of the patients.

	CHB (n = 91)	CHC (n = 108)	Control (n = 23)
Age (years old)	41.0 ± 10.4*	54.8 ± 11.2**	38.5 ± 10.3
Gender (male/female)	66/25*^,^**	49/59	11/12
AST (IU/L)	83.8 ± 88.0*	63.7 ± 41.6	N.D.
ALT (IU/L)	139.6 ± 168.1*^,^**	75.7 ± 58.7**	10.8 ± 4.3
Platelets (x 10^3^/mL)	181.4 ± 63.7	170.2 ± 65.4	N.D.
HBV DNA (Log copies/mL)	6.0 ± 2.2	N.A.	N.A.
HBeAg (positive/negative)	71/20	N.A.	N.A.
HCV genotypes (1/2)	N.A.	71/37	N.A.
HCV RNA (L/H)	N.A.	6/103	N.A.
APRI	1.53 ± 1.62	1.32 ± 1.13	N.D.
FIB-4	2.01 ± 2.03*	2.96 ± 2.07	N.D.
Liver fibrosis (F1/F2/F3/F4)	39/22/20/10	46/26/19/17	N.D.
Inflammatory activity (A1/A2/A3)	33/43/15	41/55/12	N.D.
MiR-122 level (fold)	260.5 ± 551.1*^,^**	12.2 ± 25.9	3.70 ± 8.13
WFA(+)-M2BP level (C.O.I.)	1.26 ± 1.55*^,^**	2.84 ± 3.03**	0.44 ± 0.19

Data are presented as the mean ± standard deviation.

* p<0.05 vs CHC group;

** p<0.05 vs Control group;

AST, aspartate aminotransferase; ALT, alanine aminotransferase; APRI, AST to platelet ratio index; FIB-4, Fibrosis 4; HCV RNA L, < 100 KIU/mL; HCV RNA H, ≥ 100 KIU/mL; N.A., not applicable; N.D., not done; CHB, chronic hepatitis B; CHC, chronic hepatitis C; Control, control subjects.

### Clinical and laboratory assessments

Hematological and biochemical tests were measured by standard laboratory techniques in the central laboratories of Chiba University Hospital. Cirrhosis of the liver was diagnosed by ultrasound, computed tomography, and histology.

### Measurement of hepatitis viral markers

HBV DNA was measured using a Roche Amplicor PCR assay (detection limits: 2.6 log IU/mL; Roche Diagnostics, Tokyo, Japan). HBsAg, HBeAg and anti-HCV were determined by ELISA (Abbott, Chicago, IL, USA) [19]. HCV RNA was measured by RT-PCR (COBAS AMPLICOR HCV MONITOR assay version 2.0, Roche Diagnostics, Tokyo, Japan). HCV serotyping was determined by detecting antibodies against group-specific recombinant proteins for serotypes 1 and 2 in the putative HCV NS4 protein region by an enzyme immunoassay that is commonly used in Japan [20].

### Histological analysis of the liver

Liver tissues were obtained using a 14-gauge Tru-cut biopsy needle (Baxter, Deerfield, IL, USA), or 16 or 18-gauge BARD Max-Core (Tempe, AZ, USA). The biopsy specimen was immediately fixed with 10% formalin (Wako, Tokyo, Japan) and stained with hematoxylin-eosin. Staging of liver fibrosis and grading of the activity of liver inflammation were independently assessed by at least three experienced hepatologists. Liver fibrosis staging was defined as follows: no fibrosis (F0), fibrous expansion of portal area (F1), fibrous expansion of portal area with portal-to-portal bridging fibrosis (F2), marked bridging fibrosis with occasional nodules (F3) or cirrhosis (F4). Grading of the activity of liver inflammation was defined as follows: none or minimal (A0), mild (A1), moderate (A2) or severe (A3) [21,22].

### Measurement of fibrosis markers

Serum WFA(+)-M2BP was measured using an HISCL M2BPGi assay kit (Sysmex Co., Kobe, Japan) [17]. The aspartate aminotransferase (AST) to alanine aminotransferase (ALT) ratio (AAR) was calculated using the following equation: AAR = AST/ALT. The AST to platelet ratio index (APRI) was calculated using the following equation: APRI = [AST (/35 IU/L)/platelet counts (10^3^/μL)] × 100 [23]. Fibrosis 4 (FIB-4) score was calculated using the following equation: FIB-4 = [AST (IU/L) × Age (years)]/[ALT (IU/L)^1/2^ × platelet counts (10^3^/μL)] [23].

### MiR extraction and quantification

For extraction of miRs, 300 μL of serum was used. After addition of 100 fmol of Caenorhabditis elegans miR-39 (cel-39) mimics (Qiagen, Hilden, Germany) as the spike-in control, total RNA was extracted from the serum with NucleoSpin miRNA Plasma (Macherey-Nagel GmbH & Co KG, Düren, Germany). cDNA was synthesized using a TaqMan MicroRNA Reverse Transcription kit (Applied Biosystems, Foster City, CA) with miRNA-specific primers for miR-122 and cel-39. For miR quantification, real-time PCR was conducted using gene-specific TaqMan assay kits with a StepOne Real-Time PCR System (Applied Biosystems). Real-time PCR was performed in duplicates. The relative amount of miR-122 was calculated using the 2^-ddCt^ method with cel-39 for normalization [6].

### Statistical analysis

Data were analyzed with the chi-square test, Student’s *t*-test, and Mann-Whitney’s U-test. A *p*<0.05 was considered to be statistically significant. Statistical analyses and the receiver operating characteristic (ROC) curve analysis were performed using JMP Pro 12 (SAS Institute Inc., Cary, NC, USA).

## Results

### Patients’ characteristics in the present study

The characteristics of the patients in this study are shown in Table 1. Males were 72.5%, 45.3% and 47.8% of the study population, and the median age were 40 (range 25–68), 57.5 (range 22–71) and 35 (range 24–65) years in the 91 patients chronically infected with HBV, the 108 patients chronically infected with HCV and 23 control subjects, respectively. In the HBV-positive patients, HBeAg-positive patients were predominant (78%). In HCV patients, HCV genotype 1-infected patients were predominant (66%). In control subjects, 47.8% were male, and the mean age was 38.5±10.3 years.

We also performed liver biopsy in all HBV- and HCV-infected patients in this study. Advanced liver fibrosis (F3 or F4) was observed in 30 (33%) and 36 (33%) in HBV- and HCV-infected patients, respectively (N.S.) (Table 1). Severe liver inflammation was observed in 12 (13%) and 12 (11%) in HBV- and HCV-infected patients, respectively (N.S.) (Table 1). The level of the fibrosis marker FIB-4 was significantly lower in HBV-infected patients than HCV-infected patients (p<0.05).

### Serum miR-122 levels in patients infected with HBV and HCV

The serum miR-122 levels of HBV-infected patients were higher than those of control subjects (p<0.05) (Table 1). Of interest, the serum miR-122 levels of HBV-infected patients were higher than those of HCV-infected patients (p<0.05) (Table 1).

**Fig 1:**
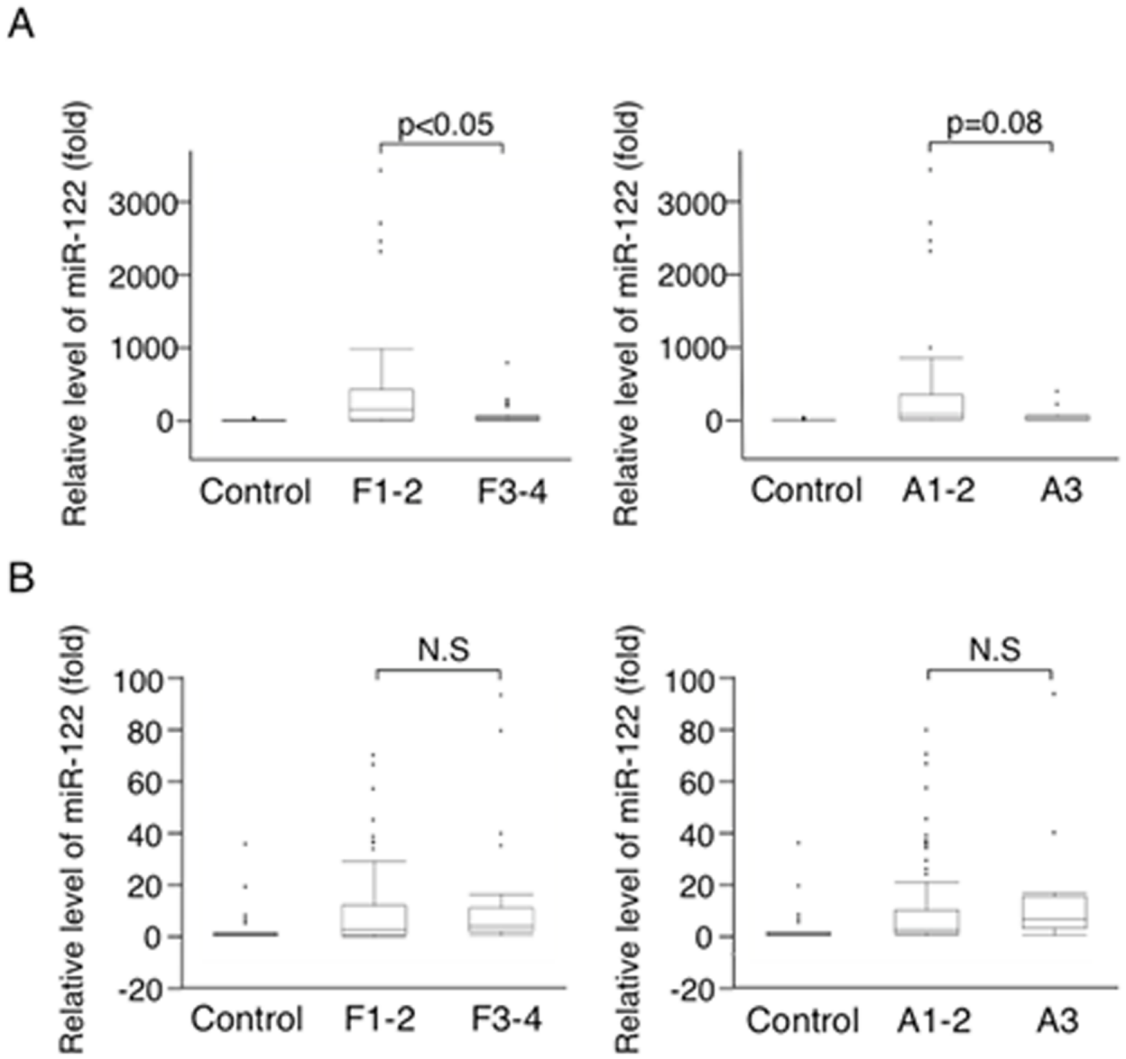
Serum miR-122 levels in the patients with HBV (A) or HCV infection (B). Liver fibrosis staging: F1, fibrous expansion of portal area; F2, fibrous expansion of portal area with portal-to-portal bridging fibrosis; F3, marked bridging fibrosis with occasional nodules; or F4, cirrhosis. Grading of the activity of liver inflammation: A1, mild; A2, moderate; or A3, severe [21,22].

In HBV-infected patients, the serum miR-122 levels of patients with severe liver fibrosis (F3 or F4) (20.3±19.0-fold) were significantly lower than those with mild or moderate liver fibrosis (F1 or F2) (192±232-fold; p<0.05). Additionally, the serum miR-122 levels of patients with severe inflammatory activity (A3) (23.7±20.6-fold) tended to be lower than those with mild or moderate (A1 or A2) (135±168-fold; p = 0.08) (Fig 1A).

On the other hand, in HCV-infected patients, the serum miR-122 levels did not differ among either the different fibrosis stages or the different inflammatory activity grades (F1 or F2, 4.70±7.14-fold vs. F3 or F4, 5.10±4.53-fold, N.S; A1 or A2, 4.04±5.60-fold vs. A3, 6.17±5.21-fold, N.S) (Fig 1B). These results suggest that lower serum miR-122 levels may be indicative of advanced liver fibrosis in HBV-infected patients.

### Serum WFA(+)-M2BP levels in patients infected with HBV and HCV

**Fig 2:**
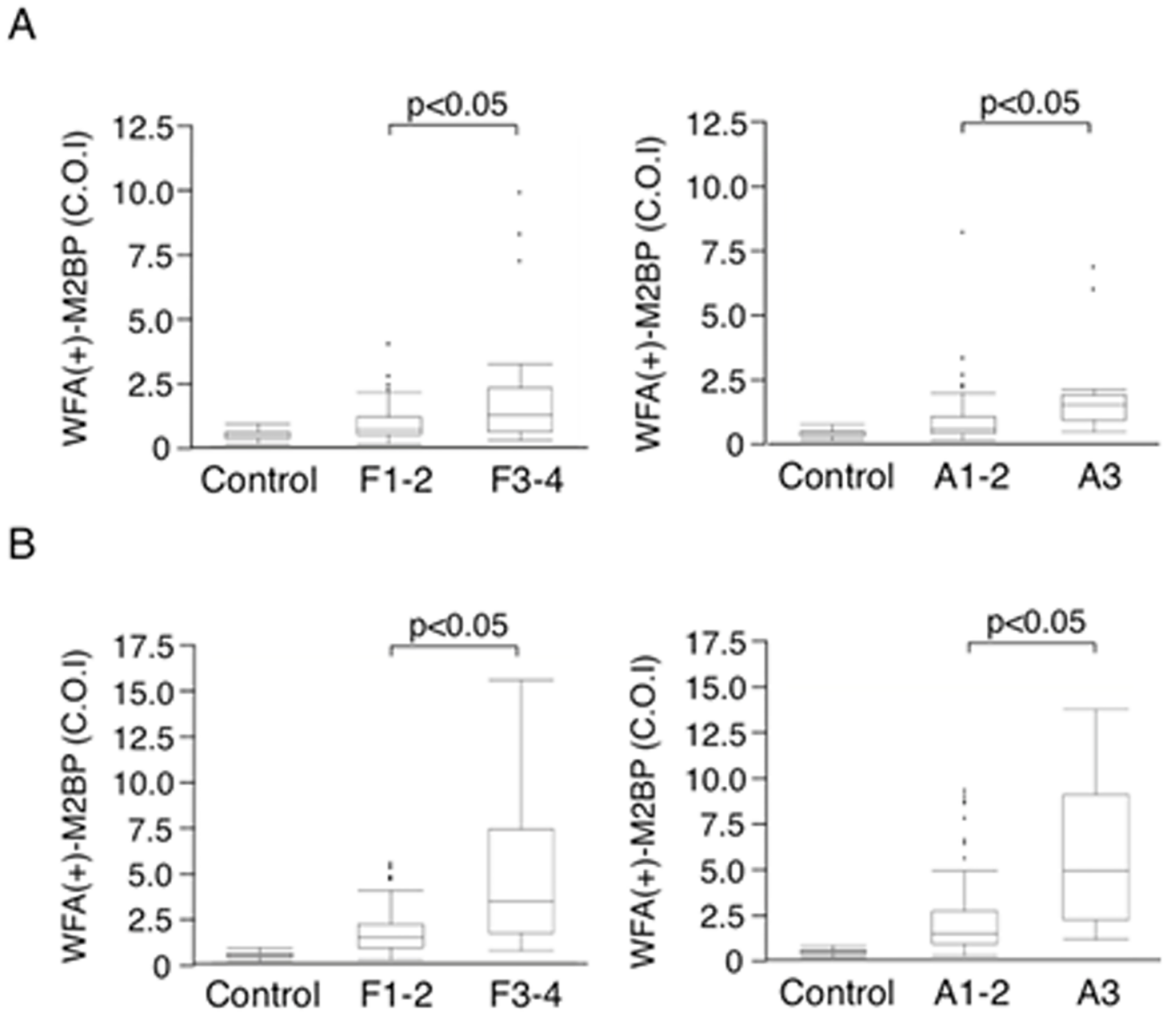
Serum WFA(+)-M2BP levels in the patients with HBV (A) or HCV infection (B). Liver fibrosis staging: F1, fibrous expansion of portal area; F2, fibrous expansion of portal area with portal-to-portal bridging fibrosis; F3, marked bridging fibrosis with occasional nodules; or F4, cirrhosis. Grading of the activity of liver inflammation: A1, mild; A2, moderate; or A3, severe [21,22].

The serum WFA(+)-M2BP levels of HBV- and HCV-infected patients were higher than those of control subjects (p<0.05) (Table 1). The serum WFA(+)-M2BP levels of HBV-infected patients were lower than those of HCV-infected patients (p<0.01) (Table 1). The serum WFA(+)-M2BP levels were higher dependent on both the staging of liver fibrosis and grading of liver inflammatory activity in HBV-infected patients (F1 or F2, 0.89±0.69 C.O.I. vs. F3 or F4, 2.0±2.3 C.O.I., p<0.05; A1 or A2, 1.1±1.3 C.O.I. vs. A3, 2.3±2.3 C.O.I., p<0.05) (Fig 2A). The serum WFA(+)-M2BP levels were higher dependent on both the staging of liver fibrosis and grading of liver inflammatory activity in HCV-infected patients (F1 or F2, 1.7±1.3 C.O.I. vs. F3 or F4, 5.1±4.1 C.O.I., p<0.05; A1 or A2, 2.1±2.1 C.O.I. vs. A3, 3.2±2.9 C.O.I., p<0.05) (Fig 2B).

### The combination of serum miR-122 and serum WFA(+)-M2BP is useful for the prediction of advanced liver fibrosis in chronic HBV infection

**Fig 3:**
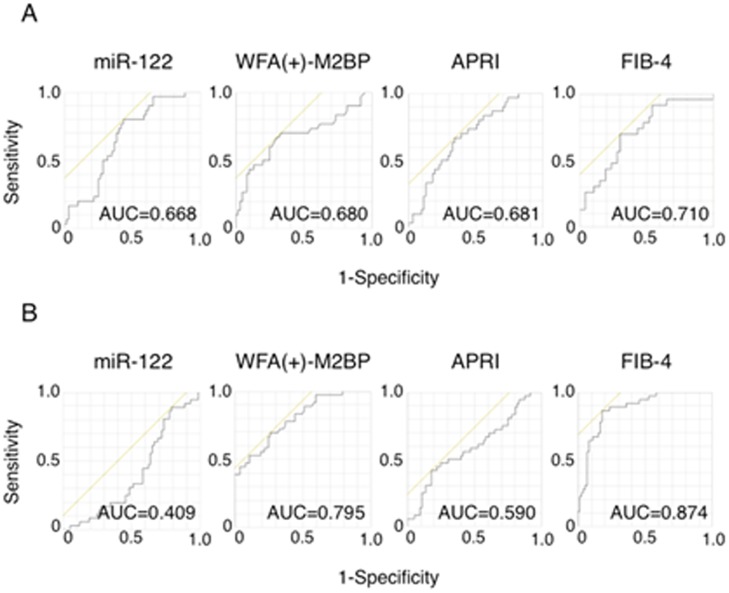
ROC curve for serum miR-122 levels, serum WFA(+)-M2BP levels, APRI and FIB-4 in the patients with HBV (A) or HCV infection (B). ROC, receiver operating characteristic; APRI, AST to platelet ratio index; FIB-4, Fibrosis 4.

The receiver operating characteristic (ROC) curve analysis was performed to assess the diagnostic ability of serum miR-122 and serum WFA(+)-M2BP for the presence of severe fibrosis. The area under the curve (AUC) of serum miR-122 for the prediction of severe fibrosis in HBV-infected patients was 0.068, which is comparable to other indexes, such as the APRI or FIB-4 index (AUC of 0.681 or 0.701, respectively). The AUC of serum WFA(+)-M2BP for the prediction of severe fibrosis in HBV-infected patients (AUC of 0.680) was almost the same as that of serum miR-122 (Fig 3A). Together, the combination of serum miR-122 and serum WFA(+)-M2BP in patients with chronic hepatitis B may have good diagnostic ability for severe fibrosis.

On the other hand, the AUC of serum miR-122 in HCV-infected patients was 0.409, which shows a lower diagnostic ability of serum miR-122 for the prediction of severe fibrosis in chronic HCV infection. In contrast, serum WFA(+)-M2BP showed a higher AUC close to that of FIB-4 index (Fig 3B). Serum WFA(+)-M2BP in patients with chronic hepatitis C possesses good diagnostic ability for severe fibrosis, as previously reported.

### Performance of serum miR-122 and serum WFA(+)-M2BP for predicting advanced liver fibrosis

In 91 HBV-infected patients, a serum miR-122 level lower than 71-fold was identified in 49 (54%) patients, and 23 of them (47%) had advanced liver fibrosis (Table 2). On the other, 42 patients with a serum miR-122 level higher than 71-fold, 7 (17%) had advanced liver fibrosis (Table 2). In the 90 HBV-infected patients, serum WFA(+)-M2BP levels higher than 1 C.O.I. augmented the prediction of advanced liver fibrosis among the HBV-infected patients with a serum miR-122 level lower than 71-fold (Table 2). However, the combination of these two factors did not augment the prediction of advanced liver fibrosis among the HCV-infected patients with serum WFA(+)-M2BP higher than 2 C.O.I. (Table 3).

**Table 2:** Predictive values for advanced liver fibrosis in patients infected with HBV.

	PPV	NPV	Sensitivity	Specificity	Accuracy
Serum miR-122 (< 71-fold) (n = 91)	47%	83%	77%	57%	64%
Serum WFA(+)-M2BP (> 1 C.O.I.) (n = 90)	53%	81%	67%	70%	69%
Serum miR-122 (< 71-fold) or serum WFA(+)-M2BP (> 1 C.O.I.) (n = 90)	44%	93%	93%	42%	59%
Serum miR-122 (< 71-fold) and serum WFA(+)-M2BP (> 1 C.O.I.) (n = 90)	64%	76%	47%	87%	74%

PPV, positive predictive value; NPV, negative predictive value

**Table 3:** Predictive values for advanced liver fibrosis in patients infected with HCV.

	PPV	NPV	Sensitivity	Specificity	Accuracy
Serum miR-122 (> 16-fold) (n = 108)	25%	65%	14%	79%	57%
Serum WFA(+)-M2BP (> 2 C.O.I.) (n = 108)	57%	83%	69%	74%	72%
Serum miR-122 (> 16-fold) or serum WFA(+)-M2BP (> 2 C.O.I.) (n = 108)	49%	82%	72%	63%	66%
Serum miR-122 (> 16-fold) and serum WFA(+)-M2BP (> 2 C.O.I.) (n = 108)	36%	67%	11%	90%	64%

PPV, positive predictive value; NPV, negative predictive value

## Discussion

Liver fibrosis assessment in liver disease, including in patients with chronic HBV infection, is an important issue [24]. Our observations suggest that the serum miR-122 levels from patients with advanced liver fibrosis were showed lower than those from patients with non-advanced liver fibrosis in HBV infection. Moreover, higher serum WFA(+)-M2BP levels augmented the prediction of advanced liver fibrosis among HBV-infected patients with lower serum miR-122 (Table 2). However, serum miR-122 levels did not differ between patients with advanced liver fibrosis and patients with non-advanced liver fibrosis in HCV infection. However, serum WFA(+)-M2BP levels from HCV-infected patients with advanced liver fibrosis showed higher values than those from HCV-infected patients with non-advanced liver fibrosis, supporting the previous study [16].

Serum WFA(+)-M2BP predicts liver fibrosis and progression to hepatocellular carcinoma (HCC) in patients with chronic HBV infection [25]. Nishikawa et al. [26] reported that the GMPH score using 4 parameters [gamma-glutamyl transpeptidase, WFA(+)-M2BP, platelet count and hyaluronic acid] is useful for the detection of advanced liver fibrosis in patients with chronic hepatitis B. The present study demonstrated that the combination of serum miR-122 and WFA(+)-M2BP could predict advanced liver fibrosis in patients with HBV infection. During the follow-up, HCC occurred in 2 patients with chronic hepatitis B. At the baseline, serum miR-122/WFA(+)-M2BP were 0.20-fold/0.4 C.O.I. (observation period: 3538 days) and 4.6-fold/0.17 C.O.I. (observation period: 6100 days), in one cirrhotic patients who was dead from HCC and one non-cirrhotic patient who is alive with HCC, respectively (Tables A-B in S1 File). As the number of patients was small in the present study, further studies will be needed to clarify whether these markers are useful for the prediction of the occurrence of HCC.

The present study also found that serum miR-122 levels from patients with severe liver inflammation showed lower values than those from patients with non-severe liver inflammation in HBV infection. Hepatic expression of miR-122 in HBV patients has been reported to not be associated with viral load or liver injury [27].

The serum miR-122 levels from patients with chronic HBV infection are higher than those from patients with chronic HCV infection and healthy controls. Several miRNAs, including miR-122, miR-22, and miR-99a, have been reported to be up-regulated at least 1.5-fold in serum of HBV-infected patients [28]. MiR-122 is involved in HBV replication [14,29] and the regulation of cell proliferation [14,30] in HBV infection. In HCV infection, miR-122 is associated with hepatocyte proliferation [31], apoptosis [32], lipid metabolism [33], interferon response [6], liver fibrosis [6], and HCV replication [12,13]. Further studies will be needed at this point.

MiR-122 and WFA(+)-M2BP are mainly produced by hepatocytes and hepatic stellate cells, respectively [13,34]. At the present time, we do not know whether WFA(+)-M2BP secreted by hepatic stellate cells is modulated by miR-122 produced by hepatocytes. In conclusion, a lower serum miR-122 level is a useful predictor of advanced liver fibrosis in HBV-infected patients. Serum WFA(+)-M2BP could also predict liver fibrosis in HBV and HCV infection. The combination of these serum markers as liquid biopsies may result in the more accurate evaluation of liver fibrosis in HBV infection. Circulating miR-122 and WFA(+)-M2BP are promising biomarkers for liver fibrosis and the prognosis of HBV-infected individuals.

## Supporting information

S1 File Patients’ characteristics in this study. Table A. Patients with chronic hepatitis B (n = 91). Table B. Patients with chronic hepatitis C (n = 108). Table C. Control subjects (n = 23).
(XLSX)
